# CircGPC3 promotes hepatocellular carcinoma progression and metastasis by sponging miR-578 and regulating RAB7A/PSME3 expression

**DOI:** 10.1038/s41598-024-58004-y

**Published:** 2024-04-01

**Authors:** Linling Ju, Yunfeng Luo, Xiaohui Cui, Hao Zhang, Lin Chen, Min Yao

**Affiliations:** 1 Medical School of Nantong University, Nantong University, Institute of Liver Diseases, Affiliated Nantong Hospital 3 of Nantong University, Nantong Third People’s Hospital,, 60 Middle Qingnian Road, Nantong, 226000 Jiangsu China; 2 Nantong Hospital Affiliated to Nanjing University of Chinese Medicine, 41 Jianshe Road, Nantong, 226009 Jiangsu China

**Keywords:** Non-coding RNAs, Oncogenes, Cell biology

## Abstract

CircRNAs are a class of highly stable noncoding RNAs that play an important role in the progression of many diseases, especially cancer. In this study, high-throughput sequencing was used to screen for abnormally expressed circRNAs, and we found that circGPC3 was overexpressed in HCC tissues. However, the underlying mechanism of circGPC3 in the development and metastasis of hepatocellular carcinoma (HCC) remains unknown. In our study, we found that circGPC3 was significantly upregulated in HCC tissues and cells and that its overexpression was positively correlated with overall survival, TNM stage and lymph node metastasis. In vivo and in vitro experiments showed that circGPC3 knockdown repressed HCC cell migration, invasion and proliferation and promoted apoptosis. Mechanistically, circGPC3 promoted HCC proliferation and metastasis through the miR-578/RAB7A/PSME3 axis. Our results demonstrate that circGPC3 contributes to the progression of HCC and provides an intervention target for HCC.

## Introduction

Hepatocellular carcinoma (HCC) is one of the most common malignant cancers worldwide, with a low early diagnosis rate, a high mortality rate and high metastasis rate^1^. The early clinical screening methods of HCC are mainly alpha-fetoprotein (AFP) determination and liver ultrasonography, but the sensitivity and specificity of AFP detection are limited, while ultrasonography largely depends on the subjective judgment of the operator, and conventional ultrasound does not provide sufficient information for determining the nature of liver lesions^2–4^. Therefore, it is urgent to identify effective biomarkers and to understand their roles in the occurrence and development of HCC.

Circular RNAs (circRNAs) are covalently closed structures without a 5′ cap and 3′ poly (A) tail^5^. CircRNAs are more stable than linear parental genes and can be used as biomarkers and therapeutic targets for many tumors^6–8^. In recent years, with the rapid development of high-throughput sequencing technology and bioinformatics analysis technology, many dysregulated circRNAs have been discovered to impact the pathogenesis of HCC. For example, the circGPR137B/miR-4739/FTO feedback loop suppresses tumorigenesis and metastasis of HCC^9^. Circ-ZEB1 promotes PIK3CA expression by silencing miR-199a-3p and affects the proliferation and apoptosis of HCC^10^. Circular RNA sequencing identified CircASAP1 as a key regulator in HCC metastasis^11^. Circular RNA circTRIM33-12 acts as the sponge of microRNA-191 to suppress HCC progression^12^. Although several HCC-related circRNAs have already been identified, the important roles of circRNAs in HCC progression still need to be fully elucidated. Numerous studies have demonstrated that circRNAs can function by regulating gene transcription, sponging miRNAs, interacting with proteins, or acting as translation templates for peptides and proteins^13–15^. The biological roles of circGPC3 in HCC and the underlying mechanisms remain largely unknown.

In this study, high-throughput sequencing was performed to analyze circRNA expression profiles in HCC and adjacent normal tissues. We discovered a novel circRNA derived from the GPC3 gene, circGPC3, which plays a critical role in the progression of HCC. Our data demonstrated that circGPC3 expression was remarkably upregulated in HCC tissues and promoted the migration, invasion and proliferation of HCC cells, suggesting that circGPC3 could serve as a useful potential biomarker for HCC diagnosis and prognosis and may be a target for treating HCC.

## Materials and methods

### Clinical tissues and cell culture

Fresh HCC tissues and matched adjacent nontumor tissues were obtained from patients who were diagnosed with primary HCC prior to any therapy at Nantong Third People’s Hospital between 2018 and 2021. The clinicopathological features of these patients are provided in Table 1. The experimental protocols of this study were approved by the Ethics Committee of Nantong Third People’s Hospital (No. EK2022023), and carried out in accordance with the Declaration of Helsinki. Written informed consent was obtained from each participant. All samples were collected and stored at − 80 °C until further use.

**Table 1:** The clinicopathological parameters of HCC patients.

Characteristics	No	circGPC3		*P* value
		Low	High	
Sex				0.196
Male	66	30	36	
Female	32	19	13	
Age				0.686
< 60	50	24	26	
≥ 60	48	25	23	
Serum AFP (ng/ml)				0.396
< 400	64	34	30	
≥ 400	34	15	19	
TNM stage				< 0.001
T1	12	8	4	
T2	12	10	2	
T3	31	20	11	
T4	43	11	32	
Lymph node metastasis				0.003
Negative	71	42	29	
Positive	27	7	20	

The human HCC cell lines Hep3B2.1-7, HuH-7, Li-7, PLC/PRF/5, SK-HEP-1 and the normal human liver cell line LO_2_ were purchased from the Chinese Academy of Science (Shanghai, China). HuH-7, PLC/PRF/5 and SK-HEP-1 cells were cultured in MEM (Gibco, USA); Hep3B2.1-7 and Li-7 cells were cultured in RPMI 1640 medium (Gibco, USA) supplemented with 10% fetal bovine serum (Gibco, USA).

### RNA fluorescence in situ hybridization (FISH)

Cy3-labeled probe sequences for circGPC3 and 18S rRNA were constructed by GenePharma (Suzhou, China). The signals of the probes were detected by a Fluorescent in Situ Hybridization Kit (Genepharma, China) according to the manufacturer’s protocol. The images were obtained using fluorescence microscopy. Nuclei were stained with DAPI.

### RNA preparation and RT‒PCR

The procedure was performed as previously described^16^. All primers are listed in Supplementary Table 1.

### Plasmid and siRNA construction

For circGPC3 overexpression plasmids, circGPC3 cDNA was synthesized and cloned and inserted into the pcDNA3.1 vector Genepharma (Suzhou, China). siRNA targeting circGPC3 and negative control siRNA were synthesized by GenePharma (Suzhou, China). Empty vector was used as the negative control. All sequences are listed in Supplementary Table 1.

### Cell migration and invasion assays

Transfected cells were seeded in the upper chambers (Millipore, Germany) with or without Matrigel-coated membranes (BD Biosciences, USA). Normal culture medium containing 20% FBS was loaded into the bottom chambers as the attractant. After 48 h of incubation, cells migrating or invading the membrane were fixed with 4% paraformaldehyde, stained with 0.1% crystal violet, and observed under a microscope (Olympus).

### Wound healing assay

Transfected PLC/PRF/5 or Hep3B2.1-7 cells were seeded at 100% density in 6-well plates with serum-free medium. A sterile 10 μL pipette tip was utilized to make a streak in the middle of the dish. The migration of cells at 0 and 24 h after wounding was observed under a microscope (Olympus, Tokyo, Japan).

### 5-Ethynyl-2′-deoxyuridine (EdU) assay

The Cell-Light EdU DNA Cell Proliferation Kit (RiboBio, Guangzhou, China) was used to assess cell proliferation activity according to the manufacturer’s protocol. All images were acquired using an Olympus IX73-FL-PH fluorescence microscope (Olympus, Tokyo, Japan).

### Colony formation assay

Cells were collected at 24 h after transfection, and 2 × 10^3^ cells were placed in 6 cm dishes and fixed with 1% paraformaldehyde after one week of incubation. The cell colonies were counted and analyzed under a light microscope after 0.1% crystal violet staining.

### Cell apoptosis assay

For the cell apoptosis assay, transfected cells were stained with an Annexin V-PE/7-AAD Apoptosis Detection Kit (BD Biosciences) according to the manufacturer’s instructions. The ratio of apoptotic cells was determined using a FACSCalibur (BD Biosciences). Data were analyzed using FlowJo software (FlowJo LLC, Ashland, OR, USA).

### Western blotting analysis

Cells were collected with RIPA buffer (Beyotime, Shanghai, China) with protease and phosphatase inhibitors. The protein concentration was determined by the BCA method. Proteins were electrophoresed using 10% SDS‒PAGE gels and then transferred onto nitrocellulose membranes (Millipore Corporation, USA). After blocking in nonfat milk, the membranes were incubated overnight at 4 °C with primary antibody and subsequently with secondary antibody for 1 h at room temperature. The antibodies used in Western blotting were as follows: HRP-conjugated-β-actin, E-cadherin, N-cadherin, vimentin, Bax, Bcl-2, and horseradish peroxidase (HRP)-conjugated goat anti-rabbit antibodies. The antibodies were purchased from Proteintech (Wuhan, China). Signals were visualized by an enhanced chemiluminescence detection system (Tanon, China), analyzed by Image Studio software and normalized to the internal control β-actin.

### Animal models

Eight 6-week-old BALB/c nude mice were chosen for tumor xenograft model development and divided into two groups (n = 4). PLC/PRF/5 cells (1 × 10^7^) stably transfected with LV-circGPC3 or LV-NC were inoculated subcutaneously into the flanks of nude mice. Tumor volume was calculated weekly, and tumor weight was measured at the end of the experiment. To investigate the role of circGPC3 in tumor metastasis, 100 μl suspensions of 5 × 10^5^ cells with stably downregulated circGPC3 or negative control PLC/PRF/5 cells were injected into the spleen of nude mice to establish the liver metastasis model. After one month, the livers were resected after euthanasia to evaluate the liver metastasis ability. All animal experiments were approved by the Animal Care Committee of Nantong University and carried out in accordance with the ARRIVE guidelines.

### Statistical analysis

All statistical analyzes were performed with GraphPad Prism 7.0 (GraphPad, CA, USA) and SPSS version 17.0 software. Student’s t test was used for comparisons between two groups. The differences among three or more groups were analyzed by one-way analysis of variance (ANOVA) and post-hoc test. The Kaplan‒Meier method was used to construct the survival curve. Multivariate Cox regression analysis was conducted to identify independent risk factors for OS. The data are presented as the mean ± SD (standard deviation). A *P* value < 0.05 was considered to indicate a significant difference.

### Reprints and permissions information

Is available at www.nature.com/reprints.

## Results

### CircGPC3 is upregulated in HCC tissues and is associated with HCC progression

**Figure 1:**
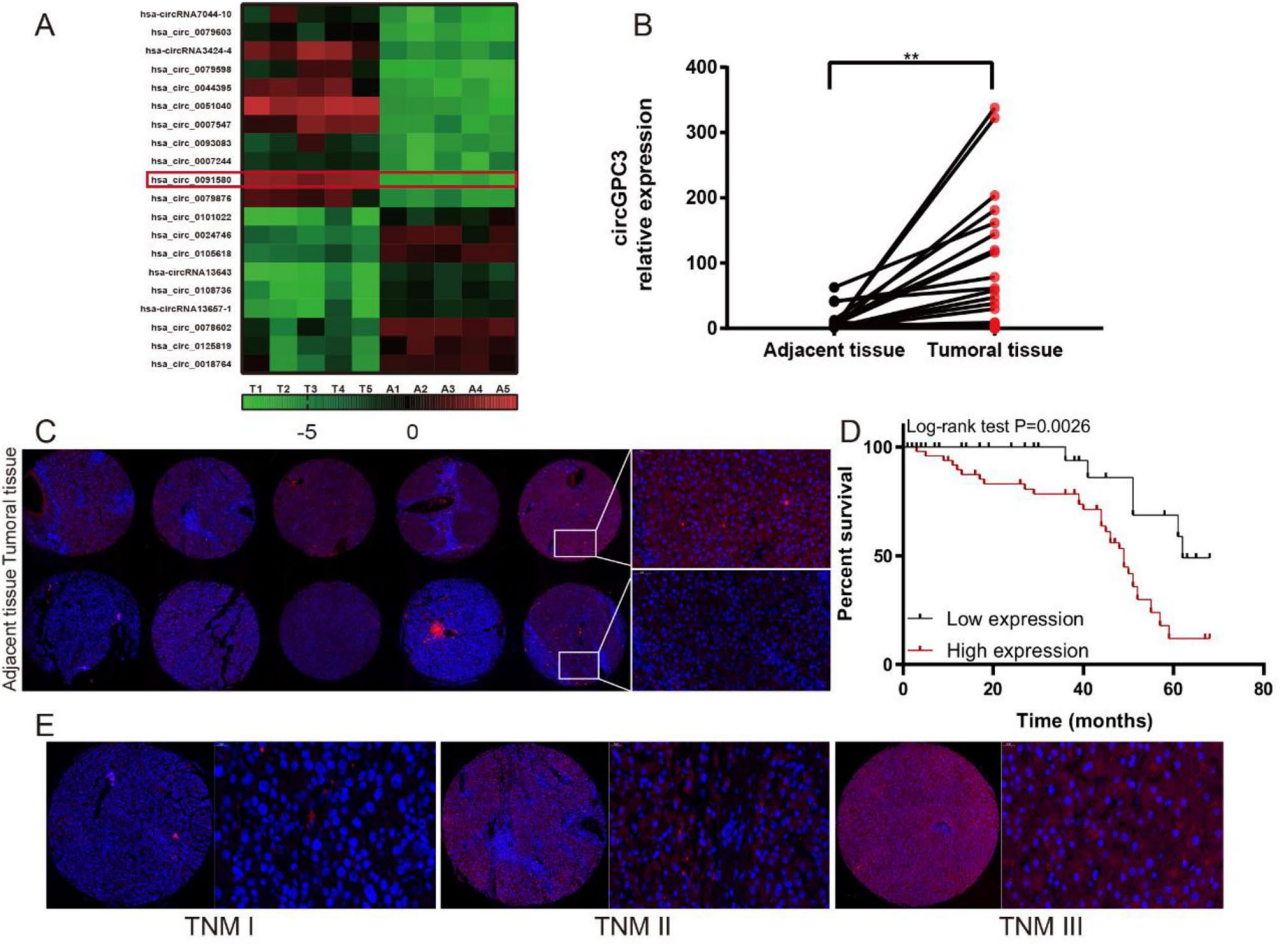
Expression and clinical significance of circGPC3 in HCC. (**A**) A circRNA microarray was used to identify the differentially expressed circRNAs between HCC and matched adjacent normal tissues. (**B**) qRT‒PCR was used to evaluate the expression of circGPC3 in paired HCC and matched adjacent normal tissues. (**C**) FISH images of circGPC3 expression in paired HCC and matched adjacent normal tissues. The nuclei were stained with DAPI (blue), and cytoplasmic circGPC3 was stained red. Right panel, scale bar 50 μm. (**D**) Kaplan–Meier analysis of the OS rate in HCC patients with high or low expression of circGPC3. (**E**) Representative FISH images of circGPC3 expression in HCC tissues with different TNM stages. **p < 0.01.

To investigate the role of circRNAs in HCC progression, we used a high-throughput human circRNA microarray to analyze the differentially expressed circRNAs in five pairs of HCC tissues and corresponding adjacent tissues. Among these significantly upregulated circRNAs, we chose circGPC3 as a candidate for further research (Fig. 1A). Using qRT‒PCR, we investigated the expression of circGPC3 in 64 pairs of HCC and adjacent tissues. The results showed that circGPC3 was significantly upregulated in HCC tissues (Fig. 1B). Furthermore, we utilized FISH assays to assess circGPC3 expression levels and found that circGPC3 expression was markedly upregulated in HCC tissues compared with adjacent normal tissues (Fig. 1C). Kaplan–Meier plots showed that the overall survival (OS) rate of patients with high circGPC3 expression was significantly lower than that of patients with low circGPC3 expression (Fig. 1D). Then, we examined the correlation of circGPC3 expression with clinicopathological findings in 98 HCC cases (Table 1). Cox regression analysis revealed that circGPC3 served as an independent risk factor for OS (Table 2). The results suggested that high expression of circGPC3 was significantly correlated with TNM stage (Fig. 1E) and lymph node metastasis but not sex, age or AFP level. Therefore, these results indicated that circGPC3 may play a vital role in HCC progression.

**Table 2:** Univariate and multivariate Cox regression analysis.

Characteristics	Hazard ratio	Univariable	*P*	Hazard ratio	Multivariable	*P*
		95% confidence interval			95% confidence interval	
circGPC3 expression	1.757	1.331–2.318	< 0.001	1.535	1.100–2.141	0.012
Gender	0.886	0.456–1.723	0.722			
TNM stage	1.448	1.061–1.978	0.020	1.053	0.720–1.542	0.789

### Identification and characteristics of circGPC3 in HCC cells

**Figure 2:**
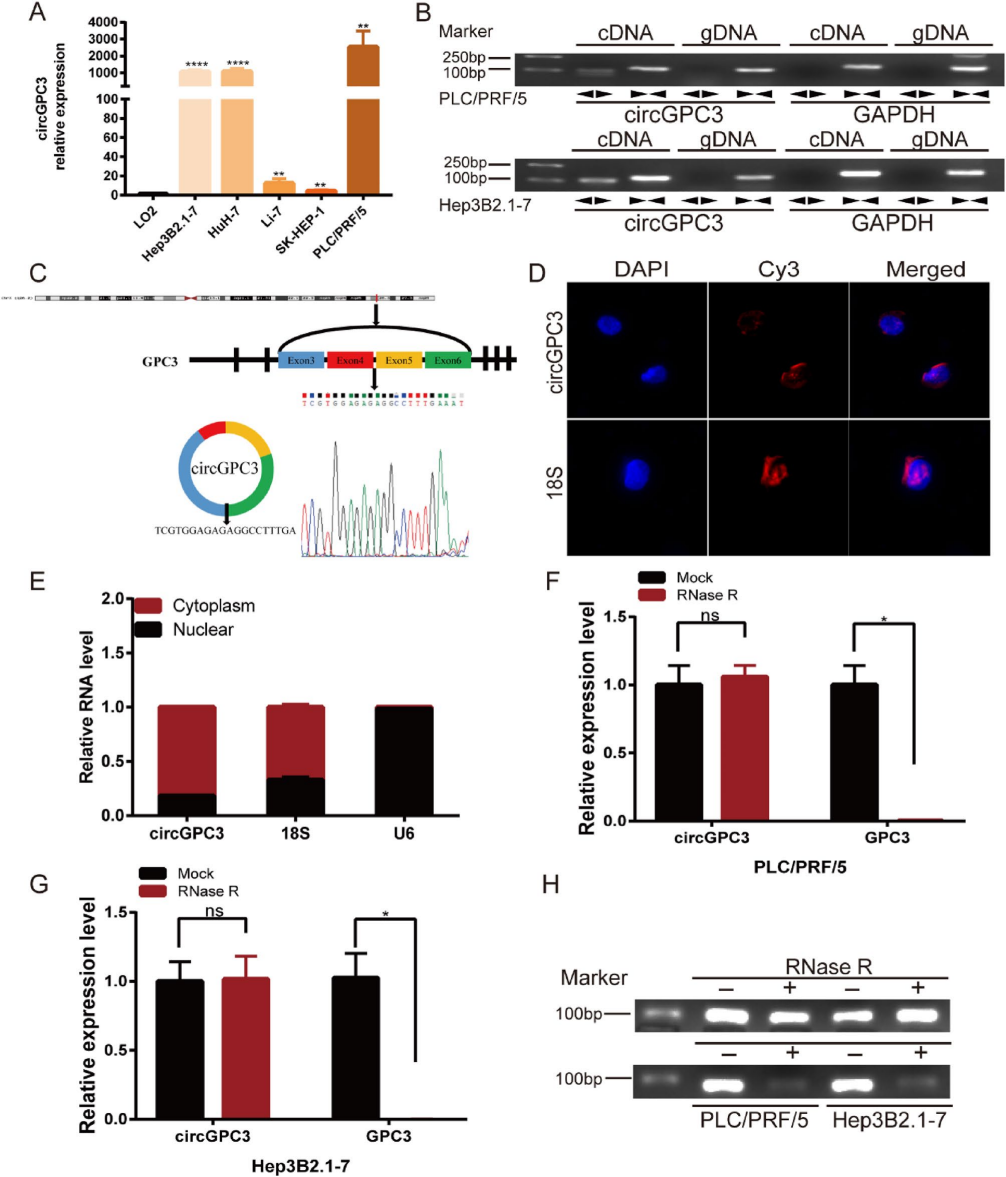
Identification of circGPC3 expression and analysis of its characteristics in HCC cells. (**A**) qRT‒PCR was used to evaluate the expression of circGPC3 in HCC cell lines and a normal liver cell line. (**B**) Convergent and divergent primers were used to amplify the back-spliced and linear products to verify the circular form of circGPC3 using PLC/PRF/5 and Hep3B2.1-7 cells. (**C**) The back-splice junction of circGPC3 was identified by Sanger sequence analysis. (**D**) FISH assay was performed to observe the subcellular localization of circGPC3. (**E**) qRT‒PCR was used to measure the expression of circGPC3 in the nucleus and cytoplasm of PLC/PRF/5 cells. (**F, G**) The expression of circGPC3 and GPC3 mRNA was measured by qRT‒PCR in PLC/PRF/5 and Hep3B2.1-7 cells treated with or without RNase R. (**H**) Verification of the circular form of circGPC3 after treatment with RNase R by agarose gel electrophoresis. *p < 0.05, **p < 0.01, ****p < 0.0001, ns, not significant.

We assessed the expression of circGPC3 in a normal liver cell line (LO2) and five HCC cell lines (Hep3B2.1-7, HuH-7, Li-7, SK-HEP-1 and PLC/PRF/5). CircGPC3 was highly expressed in HCC cell lines compared with the normal liver cell line (Fig. 2A). Except for the back-splice junction area, circRNAs share the same sequences as their linear parental genes, so we further verified the circular form of GPC3. We designed convergent and divergent primers to amplify GPC3 mRNA and circGPC3. cDNA and gDNA extracted from PLC/PRF/5 and Hep3B2.1-7 cells were used as templates. The results showed that circGPC3 was amplified by the divergent primers from cDNA but not from gDNA (Fig. 2B). CircGPC3 is generated from exons 3–6 of the GPC3 gene located on chromosome X: 132,826,396-132,888,203. Sanger sequencing analysis of the PCR products confirmed the head-to-tail splicing of circGPC3 (Fig. 2C). Moreover, we detected the subcellular localization of circGPC3 through FISH and nuclear mass separation assays. The results showed that most circGPC3 was located in the cytoplasm (Fig. 2D,E). Since high stability is one of the main features of circRNAs, we further confirmed that circGPC3 was more resistant to RNase R digestion than linear GPC3 mRNA (Fig. 2F–H). Taken together, the results demonstrated that circGPC3 was a highly stable circRNA and was predominantly localized in the cell cytoplasm.

### CircGPC3 knockdown inhibits the migration and invasion of HCC cells

**Figure 3:**
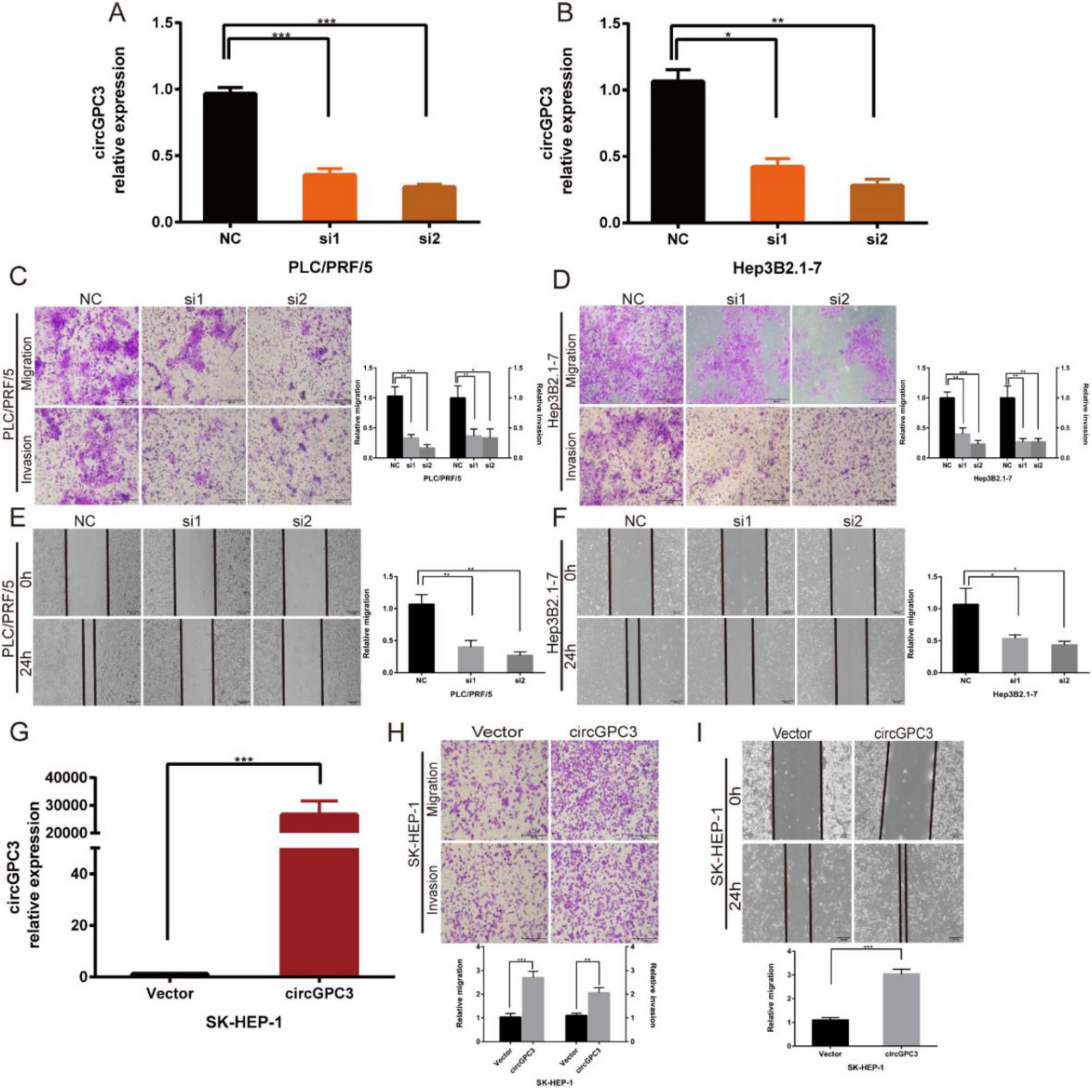
circGPC3 knockdown inhibits HCC cell migration and invasion. (**A, B**) qRT‒PCR was used to determine the expression of circGPC3 in PLC/PRF/5 and Hep3B2.1-7 cells transfected with two independent siRNAs targeting circGPC3. (**C, D**) Transwell assays were used to evaluate the migratory and invasive capabilities of PLC/PRF/5 and Hep3B2.1-7 cells when circGPC3 was downregulated. (**E, F**) Wound healing assays at 0 h and 24 h were performed to assess cell migratory capability after knocking down circGPC3 in PLC/PRF/5 and Hep3B2.1-7 cells. (**G**) qRT‒PCR was used to determine the expression of circGPC3 in SK-HEP-1 cells transfected with the circGPC3 overexpression plasmid. (**H**) Transwell assays were used to evaluate the migratory and invasive capabilities of SK-HEP-1 cells when circGPC3 was upregulated. (**I**) Cell migratory capability was assessed by the wound healing assay after upregulating the expression of circGPC3 in SK-HEP-1 cells. *p < 0.05, **p < 0.01, ***p < 0.001.

To elucidate the molecular mechanism underlying circGPC3 in the behaviors of HCC cells, siRNAs were constructed and synthesized to specifically target the back-spliced region of circGPC3 to downregulate its expression. The qRT‒PCR results demonstrated that circGPC3 expression in PLC/PRF/5 and Hep3B2.1-7 cells transfected with siRNAs was effectively decreased (Fig. 3A,B). Transwell and wound healing assays were used to detect the roles of circGPC3 in the migration and invasion of HCC cells. The results showed that circGPC3 knockdown obviously decreased the migratory and invasive capabilities of PLC/PRF/5 and Hep3B2.1-7 cells (Fig. 3C–F).

To further determine whether circGPC3 overexpression has the opposite effects, we constructed a circGPC3 overexpression plasmid. Then, the circGPC3 overexpression plasmid was stably transfected into SK-HEP-1 cells. The qRT‒PCR results indicated that circGPC3 expression was significantly upregulated after stable transfection (Fig. 3G). Functionally, Transwell and wound healing assays revealed that overexpression of circGPC3 increased the migration and invasion ability of SK-HEP-1 cells (Fig. 3H,I).

### CircGPC3 knockdown represses HCC cell proliferation but promotes apoptosis

**Figure 4:**
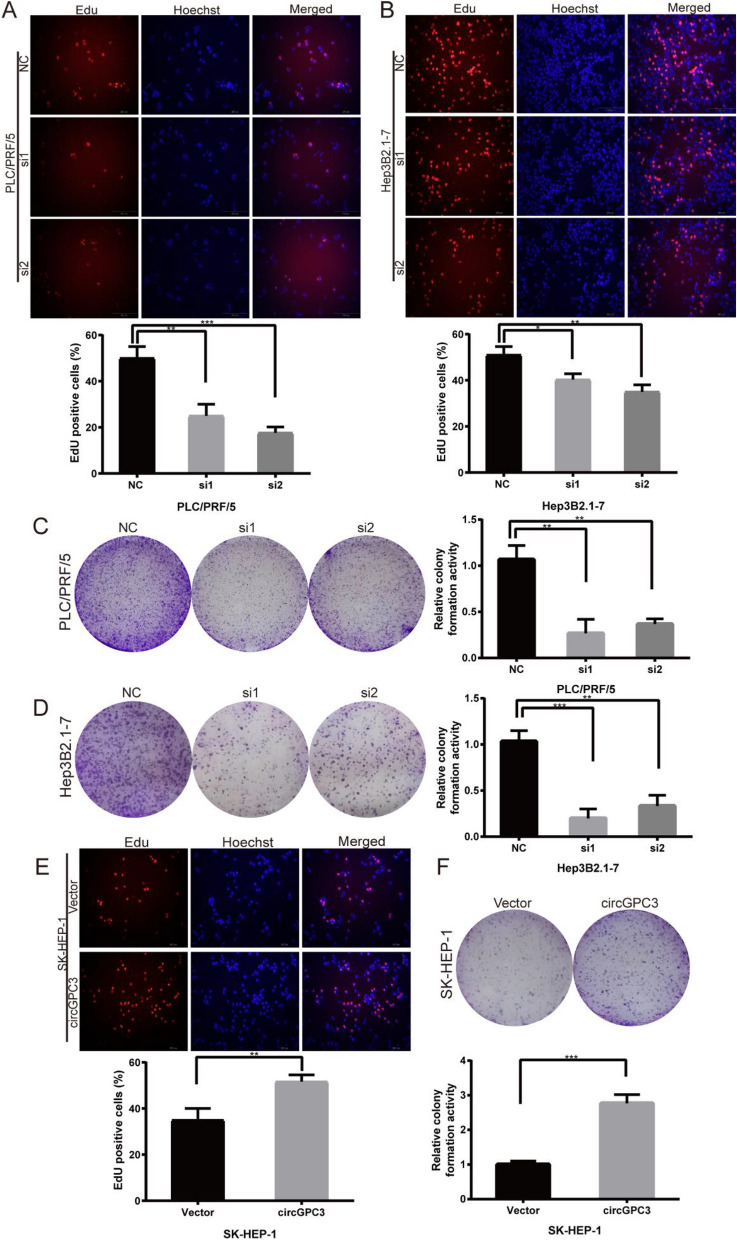
circGPC3 knockdown inhibits HCC cell proliferation. (**A, B**) EdU assays were used to evaluate the proliferative capacities of PLC/PRF/5 and Hep3B2.1-7 cells when circGPC3 was downregulated. (**C, D**) Colony formation assays were used to evaluate the proliferative capacities of PLC/PRF/5 and Hep3B2.1-7 cells. (**E**) EdU assays were used to evaluate the proliferative capacities of SK-HEP-1 cells when circGPC3 was upregulated. (**F**) Colony formation assays were used to evaluate the proliferative capacities of SK-HEP-1 cells. *p < 0.05, **p < 0.01, ***p < 0.001.

**Figure 5:**
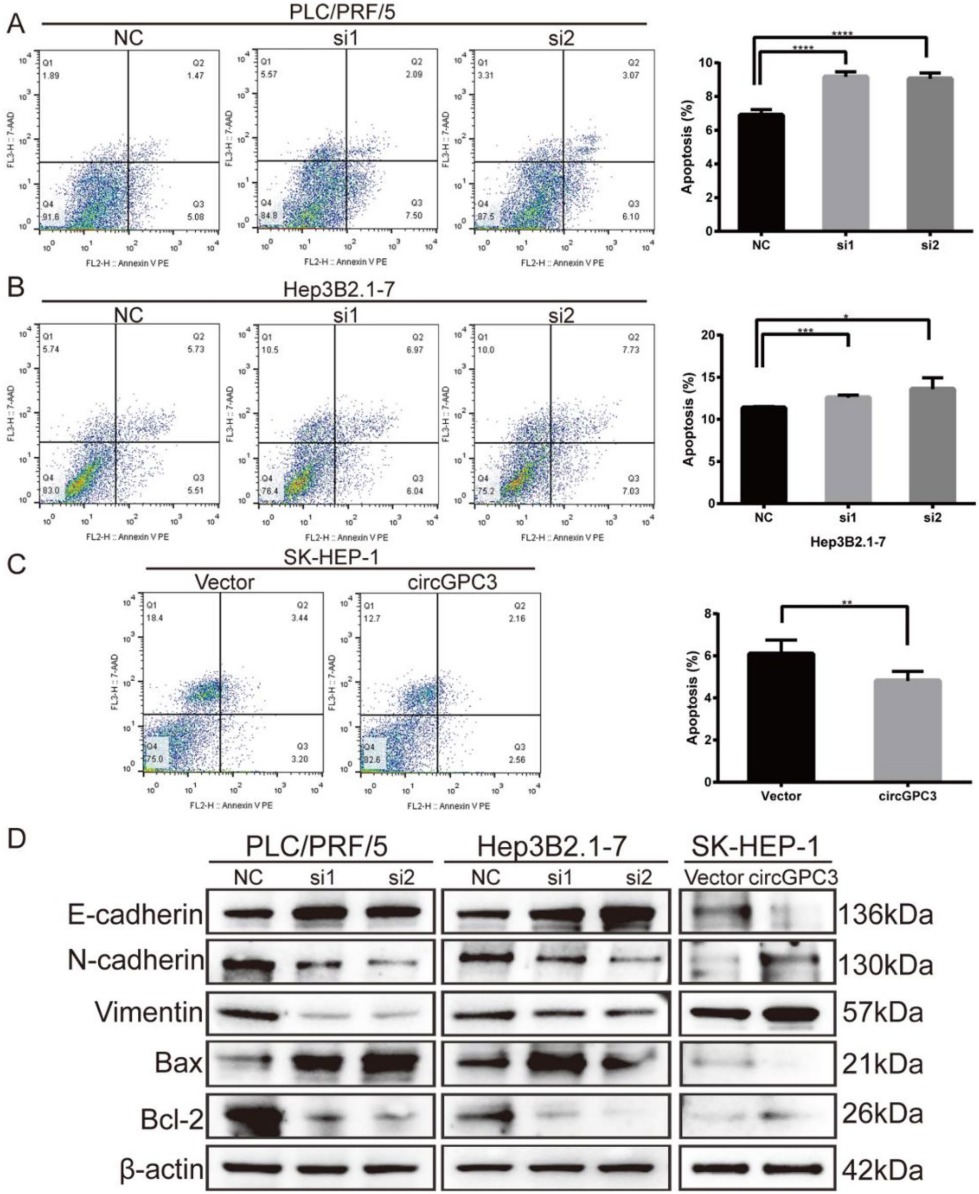
circGPC3 knockdown promotes HCC cell apoptosis. (**A, B**) Cell apoptosis was measured by flow cytometry in PLC/PRF/5 and Hep3B2.1-7 cells transfected with siRNAs. (**C**) Apoptosis in SK-HEP-1 cells was analyzed by flow cytometry when circGPC3 was upregulated. (**D**) Western blot analysis was performed to detect the protein levels of E-cadherin, N-cadherin, vimentin, Bax and Bcl-2 in PLC/PRF/5, Hep3B2.1-7 and SK-HEP-1 cells after circGPC3 knockdown or overexpression. *p < 0.05, **p < 0.01, ***p < 0.001, ****p < 0.0001.

We then investigated the effect of circGPC3 on HCC cell proliferation. The EdU assay suggested that the percentages of EdU-positive cells were greatly decreased after knockdown of circGPC3 (Fig. 4A,B). Colony formation assays showed that circGPC3 knockdown significantly inhibited colony formation ability (Fig. 4C,D). In contrast, circGPC3 overexpression exerted the opposite effects on the proliferation of HCC cells. EdU and colony formation assays confirmed that overexpression of circGPC3 enhanced cell proliferation (Fig. 4E,F). Flow cytometry demonstrated that circGPC3 knockdown promoted cell apoptosis, while circGPC3 overexpression suppressed apoptosis (Fig. 5A–C). We further monitored the changes in key proteins of the EMT process and apoptosis pathway induced by circGPC3 knockdown and overexpression. Western blot analysis showed that circGPC3 knockdown increased the expression levels of E-cadherin and Bax while decreasing the expression levels of N-cadherin, vimentin and Bcl-2. Conversely, circGPC3 overexpression induced the opposite effects (Fig. 5D).

### CircGPC3 knockdown suppresses HCC growth and metastasis in vivo

**Figure 6:**
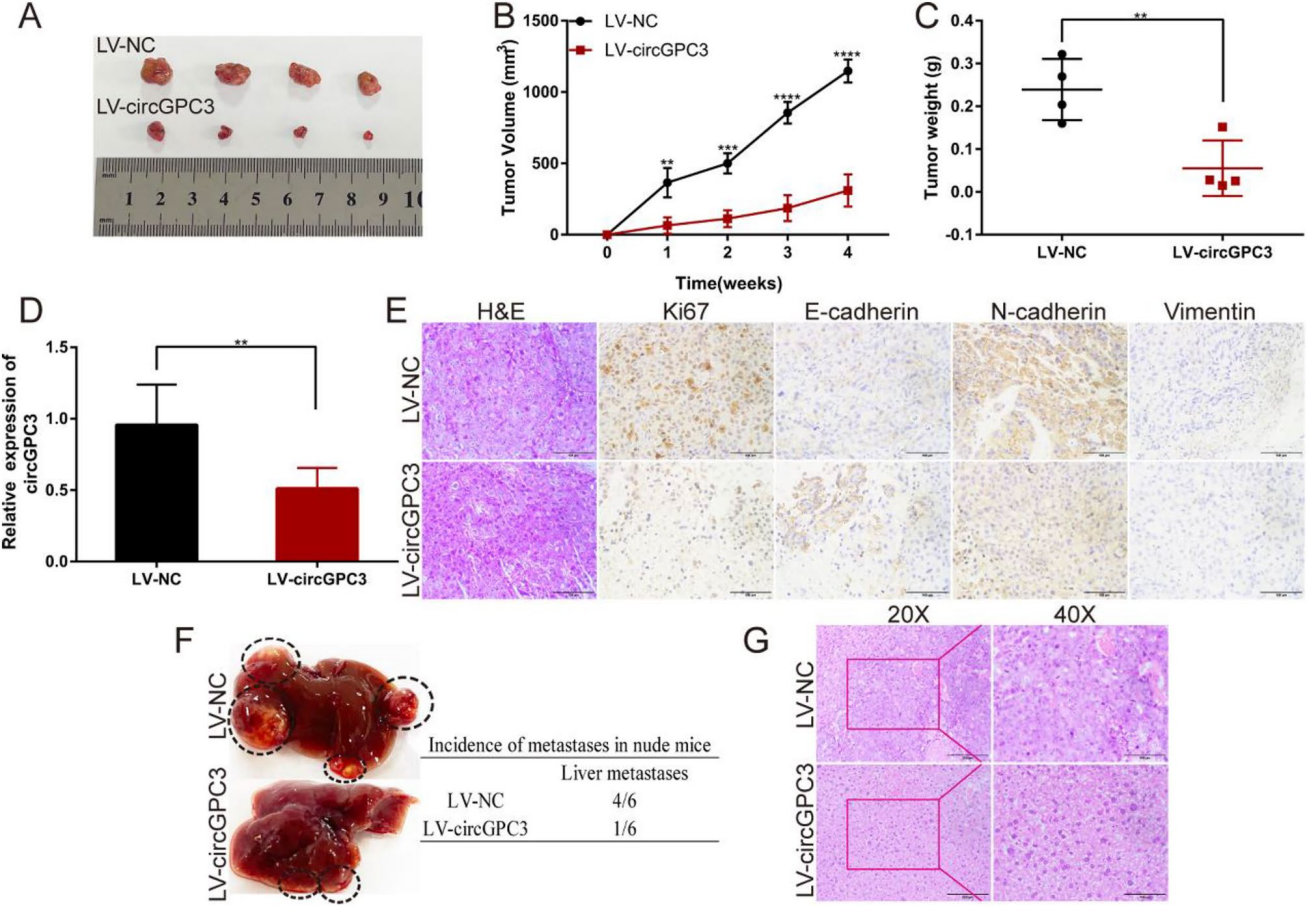
circGPC3 knockdown suppresses HCC growth and metastasis in vivo. (**A**) Image of subcutaneous tumors in nude mice after PLC/PRF/5 cell injection. (**B**) The volume of tumors in nude mice was calculated weekly for 4 weeks. (**C**) The weight of tumors was recorded after dissection of the tumors. (**D**) qRT‒PCR was used to detect the expression of circGPC3 in both the LV-circGPC3 and LV-NC groups. (**E**) H&E stained images of transplanted tumor tissues and IHC stained images of tumor tissues stained with Ki-67, E-cadherin, N-cadherin and vimentin. Scale bar, 100 μm. (**F**) Images of liver metastasis in nude mice after injection of PLC/PRF/5 cells stably transfected with LV-circGPC3 and LV-NC vectors into the spleen. (**G**) H&E stained images of micrometastases. Scale bar: left, 200 μm; right, 100 μm. **p < 0.01, ***p < 0.001, ****p < 0.0001.

To determine the effect of circGPC3 on HCC growth and metastasis in vivo, we established subcutaneous transplanted tumor and liver metastasis models in nude mice. PLC/PRF/5 cells transfected with stable circGPC3 knockdown lentiviral vectors (LV-circGPC3) and negative control lentiviral vectors (LV-NC) were inoculated subcutaneously into the axilla of nude mice. The size, volume and weight of tumors formed by cells with LV-circGPC3 were significantly smaller than those in the LV-NC group (Fig. 6A–C). qRT‒PCR analysis verified the lentiviral transfection efficiency in transplanted tumor tissues, and the results showed that the expression of circGPC3 was reduced in the LV-circGPC3 group compared with the LV-NC group (Fig. 6D). H&E staining of transplanted tumor tissues showed typical tumor morphological features. IHC staining suggested that the expression of Ki67, N-cadherin and vimentin was significantly increased in the tumors derived from cells with LV-circGPC3 compared to the control (Fig. 6E). PLC/PRF/5 cells transfected with LV-circGPC3 or LV-NC vector were injected into the spleen to construct the liver metastasis model to study the role of circGPC3 in liver metastasis. The results showed that there were fewer metastatic foci in the livers of nude mice in the LV-circGPC3 group than in the LV-NC group (Fig. 6F). H&E staining was used to detect micrometastases (Fig. 6G). These findings revealed that circGPC3 knockdown suppressed the growth and metastasis of HCC in vivo.

### CircGPC3 regulates HCC growth and metastasis by sponging miR-578

**Figure 7:**
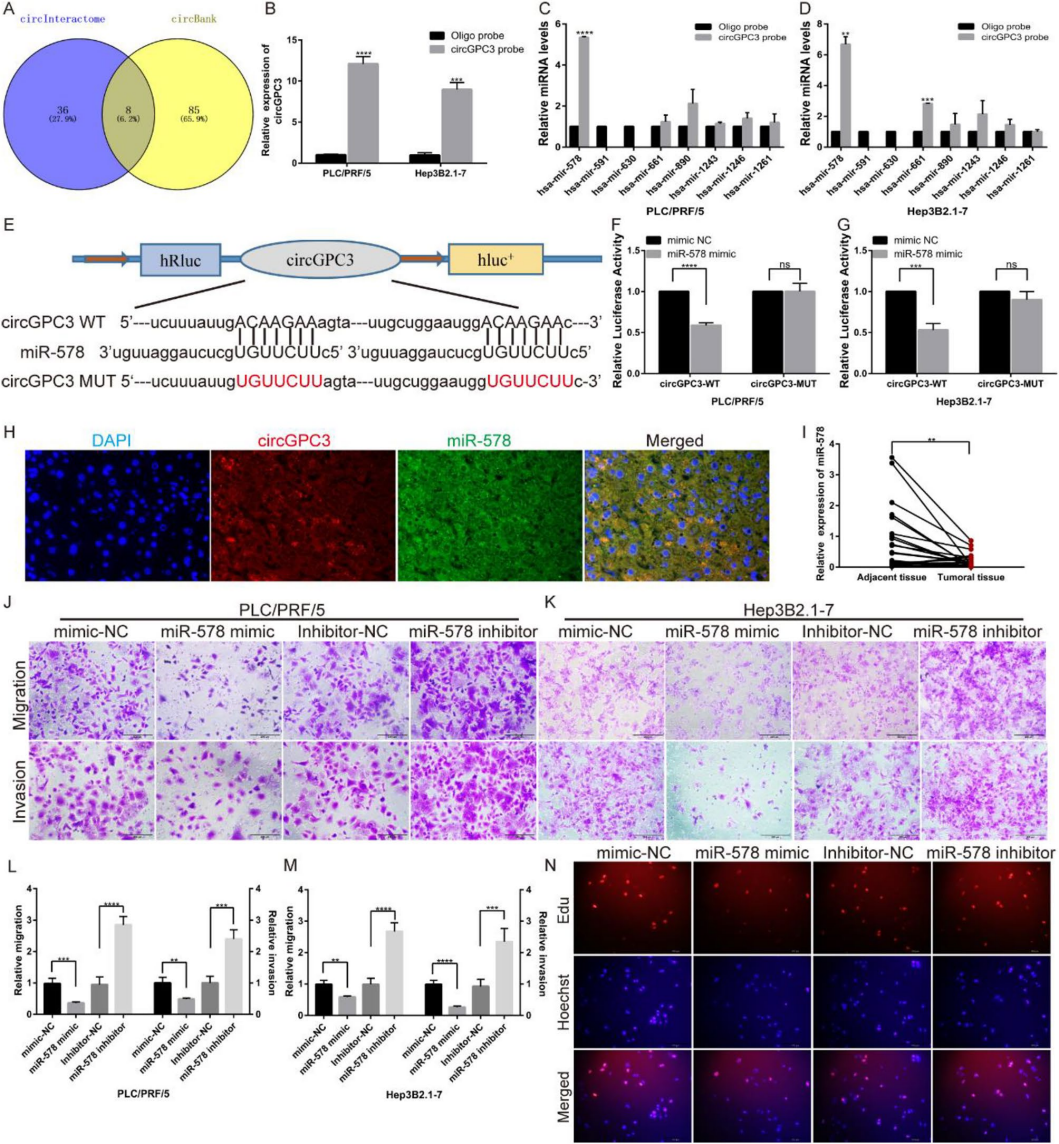
CircGPC3 regulates HCC growth and metastasis by sponging miR-578. (**A**) Venn diagrams showing overlapping miRNAs that bind to circGPC3 identified by circBank and circInteractome. (**B**) CircGPC3 biotin-labeled probes could capture more circGPC3. (**C, D**) In the RNA pull-down assay, qRT‒PCR analysis was performed on PLC/PRF/5 and Hep3B2.1-7 cells to predict circGPC3-target miRNAs. (**D**) Schematic illustration of the binding sites of circGPC3 and miR-578. (**E–G**) Relative luciferase activities were calculated in PLC/PRF/5 and Hep3B2.1-7 cells cotransfected with miR-578 mimics or mimic-NC and circGPC3-WT or circGPC3-MUT vectors. (**H**) FISH assay was used to observe the subcellular localization of circGPC3 (red) and miR-578 (green). (**I**) qRT‒PCR was performed to evaluate the expression of miR-578 in HCC and adjacent normal tissues. (**J, K**) Transwell assays were used to evaluate the migratory and invasive capabilities of PLC/PRF/5 and Hep3B2.1-7 cells by transfecting these cells with miR-578 mimics or inhibitors. (**L, M**) Relative migration and invasion rates of PLC/PRF/5 and Hep3B2.1-7 cells were calculated for cells transfected with miR-578 mimics or inhibitors. (**N**) EdU assays were used to evaluate the proliferative capacities of PLC/PRF/5 cells after transfection with miR-578 mimics or inhibitors. **p < 0.01, ***p < 0.001, ****p < 0.0001, ns, not significant.

We used circBank and circInteractome, two network tools for exploring the interactions between circRNAs and miRNAs, to predict the binding sites of circGPC3. We predicted 8 miRNA binding sites in the circGPC3 sequence (Fig. 7A). CircGPC3 biotin-labeled probes and oligo control probes were designed for an RNA pull-down assay. The RNA pull-down assay was applied to test the possible circGPC3-miRNA interactions. The qRT‒PCR results suggested that circGPC3 biotin-labeled probes were significantly enriched with miR-578 in both PLC/PRF/5 and Hep3B2.1-7 cells (Fig. 7B–D). Moreover, circGPC3 fragments containing the binding site or mutation site of miR-578 were cloned and inserted into luciferase reporter plasmids (Fig. 7E). A dual-luciferase reporter assay confirmed the direct binding of circGPC3 to miR-578 in both PLC/PRF/5 and Hep3B2.1-7 cells (Fig. 7F,G). FISH assays were performed to detect the subcellular localization of circGPC3 and miR-578. The results showed that circGPC3 and miR-578 were colocalized in the cytoplasm (Fig. 7H). Next, we explored the effect of miR-578 on HCC growth and metastasis. In HCC tissues, the qRT‒PCR results showed that the expression of miR-578 was significantly downregulated compared to that in adjacent tissues (Fig. 7I). We investigated the biological functions by transfecting miR-578 mimics or inhibitors into HCC cells. Functionally, miR-578 mimics significantly inhibited the migration, invasion and proliferation of HCC cells (Fig. 7J–N).

**Figure 8:**
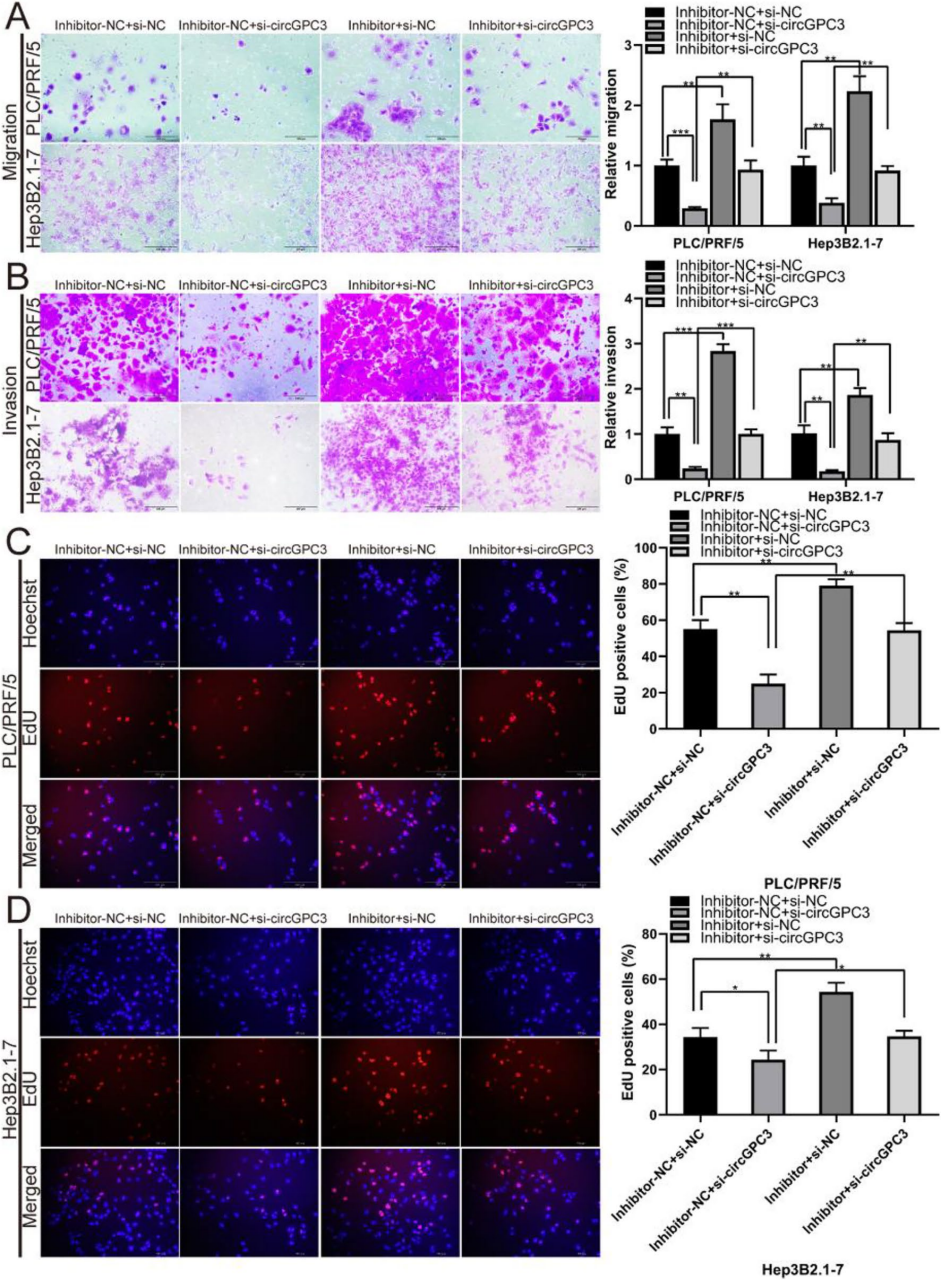
miR-578 rescues the tumor-inhibition effects caused by circGPC3 knockdown in HCC cells. (**A, B**) Transwell assays were used to evaluate the migratory and invasive capabilities of PLC/PRF/5 and Hep3B2.1-7 cells cotransfected with si-circGPC3 or si-NC and miR-578 inhibitor or inhibitor-NC. (**C, D**) EdU assays were used to evaluate the proliferative capacities of PLC/PRF/5 and Hep3B2.1-7 cells cotransfected with si-circGPC3 or si-NC and miR-578 inhibitor or inhibitor-NC. *p < 0.05, **p < 0.01, ***p < 0.001.

To further confirm the relationship between circGPC3 and miR-578 in HCC growth and metastasis, rescue assays of si-circGPC3 and miR-578 inhibitor cotransfection were performed in PLC/PRF/5 and Hep3B2.1-7 cells. The results of Transwell and EdU assays showed that circGPC3 knockdown inhibited the migration, invasion and proliferation abilities of PLC/PRF/5 and Hep3B2.1-7 cells, while miR-578 inhibitors eliminated the inhibitory effects caused by circGPC3 knockdown (Fig. 8A–D). Taken together, these results demonstrated that circGPC3 plays an important role in HCC progression by binding to miR-578.

### CircGPC3 regulates RAB7A/PSME3 expression through miR-578

**Figure 9:**
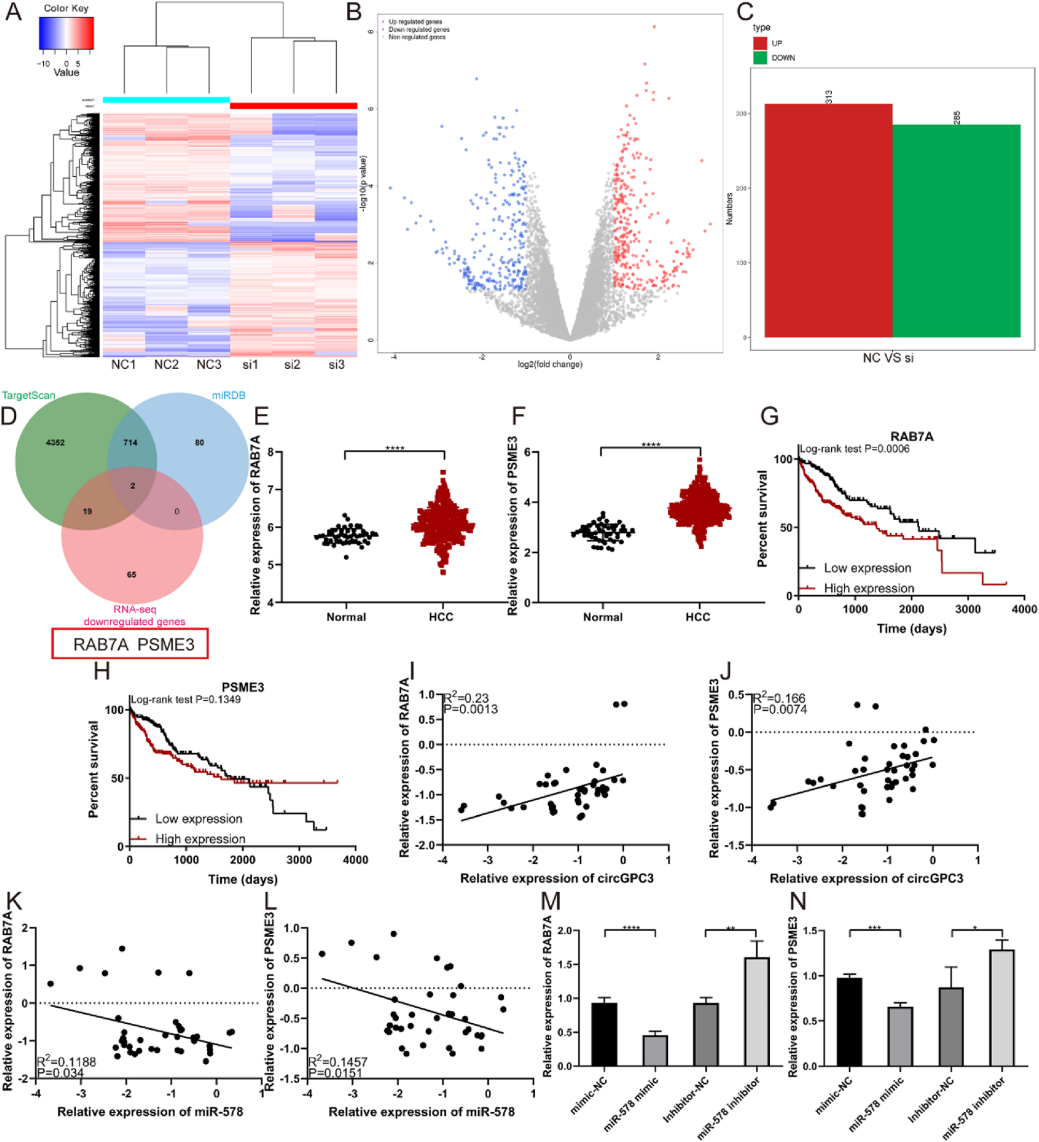
CircGPC3 regulates RAB7A/PSME3 expression through miR-578. (**A**) Heatmap of differentially expressed genes in PLC/PRF/5 cells transfected with si-NC and si-circGPC3. The analysis was performed using the R package DESeq (version 1.28.0). (**B**) Volcano plots of differentially expressed genes. (**C**) Differentially expressed upregulated and downregulated genes identified by RNA sequencing. (**D**) A Venn diagram was used to screen the potential target genes by TargetScan, miRDB and RNA-seq. (**E, F**) Expression levels of RAB7A and PSME3 in HCC based on TCGA samples. (**G, H**) Kaplan–Meier analysis of the OS rate in HCC patients with high or low expression of RAB7A and PSME3. (**I, J**) Correlation analysis of RAB7A and PSME3 expression with circGPC3 expression. (**K, L**) Correlation analysis of RAB7A and PSME3 expression with miR-578 expression. (**M, N**) qRT‒PCR was used to detect the expression levels of RAB7A and PSME3 in HCC cells transfected with miR-578 mimic or inhibitor. **p < 0.01, ***p < 0.001, ****p < 0.0001.

To investigate the molecular mechanism of circGPC3, we conducted RNA-sequencing analysis to explore its downstream potential target genes. The variation in differentially expressed genes between the si-NC group and the si-circGPC3 group was assessed by heatmaps (Fig. 9A) and volcano plots (Fig. 9B). The RNA-sequencing results showed that compared with the si-NC group, the si-circGPC3 group had 313 upregulated genes and 285 downregulated genes (Fig. 9C). TargetScan and miRDB databases were used to identify potential target genes for miR-578. By intersecting the target genes with RNA-sequencing downregulated genes, we screened out two potential target genes (RAB7A and PSME3) for further investigation (Fig. 9D). In the TCGA database, the expression levels of RAB7A and PSME3 were upregulated in HCC tissues compared with adjacent normal tissues (Fig. 9E,F). Kaplan–Meier analysis showed that patients with high RAB7A expression had a significantly lower OS rate than patients with low RAB7A expression (Fig. 9G). However, there was no significant difference in OS rates between patients with high PSME3 expression and those with low PSME3 expression (Fig. 9H). Moreover, Spearman correlation analysis showed that the expression levels of RAB7A and PSME3 in HCC tissues were positively correlated with the expression level of circGPC3 (Fig. 9I,J) and negatively correlated with the expression level of miR-578 (Fig. 9K,L). The expression levels of RAB7A and PSME3 in HCC cells transfected with miR-578 mimic or inhibitor were assessed by qRT‒PCR. The results showed that the miR-578 mimic suppressed the expression of RAB7A and PSME3, while the miR-578 inhibitor promoted the expression of RAB7A and PSME3 (Fig. 9M,N). Collectively, these results indicate that the RAB7A and PSME3 genes can be downstream target genes for miR-578 and are upregulated by circGPC3-mediated sponging of miR-578.

## Discussion

In the past two decades, with the development of medicine, the overall survival rate of HCC patients has improved significantly. At present, most drugs that have therapeutic effects also have serious adverse effects^17–19^. Therefore, there is an urgent need to develop new therapeutic strategies to treat HCC patients. More studies have found that circRNAs have an important impact on the development and metastasis of cancer^20–22^. However, the mechanism of circRNAs in HCC metastasis needs to be further studied.

circRNAs contain multiple miRNA binding sites, which can specifically sponge miRNAs to act as competitive endogenous RNAs (ceRNAs) and sequester miRNAs from target mRNAs, thereby affecting the negative regulation of target mRNAs by miRNAs^23,24^. With the in-depth study of circRNAs as miRNA sponges, researchers have tried to design in vitro synthetic circRNAs for delivery to disease models and explore the potential of circRNA drug development. For example, Roger S-Y Foo et al. designed an artificial circRNA sponge targeting miRNA (circmiR) and confirmed through in vitro and in vivo experiments that circmiR can alleviate disease progression and improve cardiac function in mouse models of heart disease by decreasing miRNA activity^25^. Honghong Yao, et al. constructed engineered rabies virus glycoprotein-circSCMH1-extracellular vesicles to deliver circSCMH1 to the brain to promote functional recovery of rodent and nonhuman primate ischemic stroke models^26^. CircRNAs are expected to replace miRNAs or linear RNAs as candidate therapies and realize the translation of basic research into clinical practice.

In previous studies, we have identified a circRNA and elucidated its roles in the development of HCC^16^. In this study, we analyzed the differentially expressed circRNAs in five pairs of HCC tissues and corresponding adjacent tissues using a high-throughput human circRNA microarray. We focused on a novel circRNA, circGPC3, which is dramatically upregulated in HCC tissues and cells. Han et al. found that circGPC3 was abnormally expressed in HCC through the clinicopathological-associated regulatory network of dysregulated circRNAs in HCC, which was consistent with our findings^27^. Clinical data analysis showed that high expression of circGPC3 was significantly correlated with TNM stage and lymph node metastasis but not sex, age or AFP level. Survival curve analysis suggested that circGPC3 was a good prognostic marker for HCC. To explore the molecular mechanism of circGPC3 in HCC, the effects of circGPC3 on the migration, invasion, proliferation and apoptosis of HCC cells were analyzed. In vitro and in vivo experiments revealed that circGPC3 promoted the growth and metastasis of HCC and may be related to the EMT process.

Then, we confirmed that miR-578 was a downstream target of circGPC3 that regulated HCC growth and metastasis. Numerous studies have shown that miRNAs play vital roles in cancer progression. MiR-578 has been reported to be involved in various cancers, such as breast cancer^28–30^, lung cancer^31,32^, osteosarcoma^33–36^ and HCC^37,38^. Our results showed that miR-578 was downregulated in tumor tissues and acted as a potent tumor suppressor. MiR-578 might suppress HCC progression, but its roles in HCC need to be further explored. RAB7A and PSME3 were confirmed to be downstream target genes coregulated by circGPC3 and miR-578. RAB7A is a small GTPase that is essential for cell migration and mitophagy^39,40^. It has been reported that RAB7A can regulate the phosphorylation state and assembly of vimentin to influence cell migration^41^. PSME3, the regulatory subunit of the proteasome, is an important oncogenic factor expressed in a variety of cancers, such as ovarian, breast and pancreatic cancers^42–44^. Our present results showed that RAB7A and PSME3 are overexpressed in HCC, and the overexpression of RAB7A and PSME3 is associated with a poorer prognosis in HCC. This finding suggests that circGPC3 could promote the progression of HCC by regulating the miR-578/RAB7A/PSME3 axis.

## Conclusions

In summary, this is the first time that circGPC3 has been reported to be significantly upregulated in HCC tissues and involved in promoting HCC progression. Mechanistically, circGPC3 promotes HCC progression through the miR-578/RAB7A/PSME3 axis. Our study provides a theoretical basis for elucidating the pathogenesis of HCC and for the targeted treatment of the disease.

### Supplementary Information

Supplementary Tables.

Supplementary Figures.

## Data Availability

The RNA-seq data used in this study are available in PRJCA021684 of BioProject.
