# Supplementary material for: CircGPC3 promotes hepatocellular carcinoma progression and metastasis by sponging miR-578 and regulating RAB7A/PSME3 expression
Source: Sci Rep. 2024 Apr 1;14:7632. doi: 10.1038/s41598-024-58004-y (PMC10984923; doi:10.1038/s41598-024-58004-y)
Supplement: Supplementary file 1 — Supplementary Tables. [file 41598_2024_58004_MOESM1_ESM.pdf]

CircGPC3 promotes hepatocellular carcinoma progression and metastasis by sponging miR-578 and regulating RAB7A/PSME3 expression

Linling Ju[1,3], Yunfeng Luo[1,3], Xiaohui Cui[1,3], Hao Zhang[2,3], Lin Chen[1*] & Min Yao[1*]

[1]Medical School of Nantong University, Nantong University, Institute of Liver Diseases, Affiliated Nantong Hospital 3 of Nantong University, Nantong Third People's Hospital, 60 Middle Qingnian Road, Nantong 226000, Jiangsu, China. [2]Nantong Hospital Affiliated to Nanjing University of Chinese Medicine, 41 Jianshe Road, Nantong 226009, Jiangsu, China. [3]These authors contributed equally: Linling Ju, Yunfeng Luo, Xiaohui Cui and Hao Zhang. ✉email: erbei@ntu.edu.cn; xiaobei227@sina.com

Supplementary Table 1. Sequences of the FISH probes.

Gene name	Probe (5'-3')
circGPC3 (FISH)	Cy3-ACAAT+TTCAA AGGCCTCTC+T CCACGAGT+TC TT
miR-578 (FISH)	FAM-ACAATCCTAGAGCACAAGAAG
18srRNA (FISH)	Cy3-CTTCCTTGGATGTGGTAGCCGTTTC

Supplementary Table 1. Sequences of the qRT–PCR primers and siRNAs.

Gene name	Forward primer (5'-3')	Reverse primer (5'-3')
circGPC3 (divergent)	TCTGCAGCCATAGCCCTGTG	TTGTGGAGTCAGGCTTGGGT
circGPC3 (convergent)	GCCCATTCTCAACAACGCCA	AGTTCCCTTCTTCGGCTGGA
RAB7A	ATTCTGGAGTCGGGAAGACATCAC	GCCTGTCATCCACCATCACCTC
PSME3	TCCCAGTCCCTGACCCCATTC	AGGCTTCTTCACACTCATCCAACC
18srRNA	GTAACCCGTTGAACCCCATT	CCATCCAATCGGTAGTAGCG
β-actin	GAGAAATCTGGCACCACACC	GGATAGCACAGCCTGGATAGCAA
GAPDH	AAGGTCGGAGTCAACGGATTTG	CCATGGGTGGAATCATATTGGAA
GAPDH (divergent)	GAAGGTGAAGGTCGAGTC	GAAGATGGTGATGGGATTTC
circGPC3 siRNA1	CGUGGAGAGAGGCCUUUGATT	UCAAAGGCCUCUCUCCACGAG
circGPC3 siRNA2	GGAGAGAGGCCUUUGAAAUTT	AUUUCAAAGGCCUCUCUCCAC
