# Supplementary figures and images for: CircGPC3 promotes hepatocellular carcinoma progression and metastasis by sponging miR-578 and regulating RAB7A/PSME3 expression
Source: Sci Rep. 2024 Apr 1;14:7632. doi: 10.1038/s41598-024-58004-y (PMC10984923; doi:10.1038/s41598-024-58004-y)

PLC/PRF/5

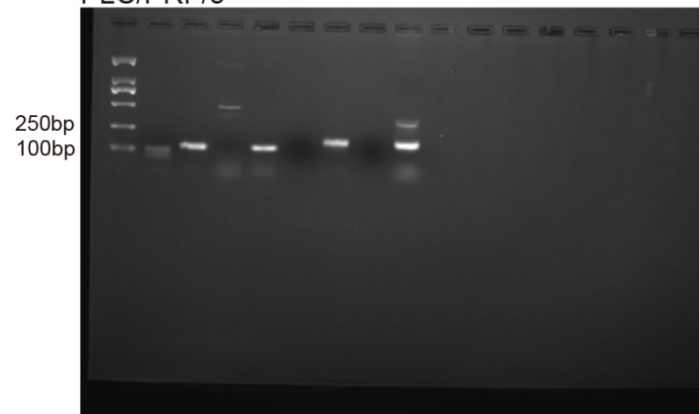

Hep3B2.1-7

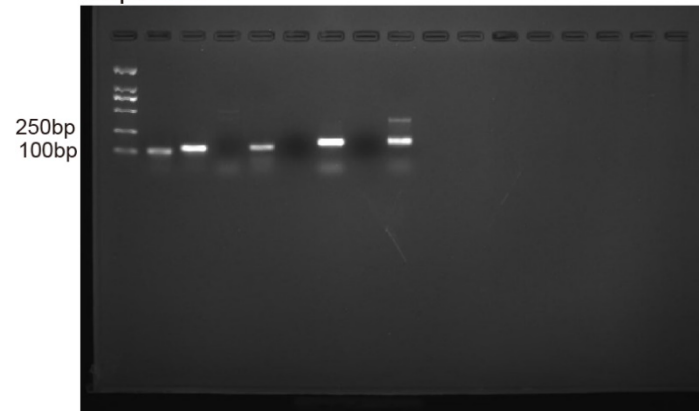

RNase R

- + - +

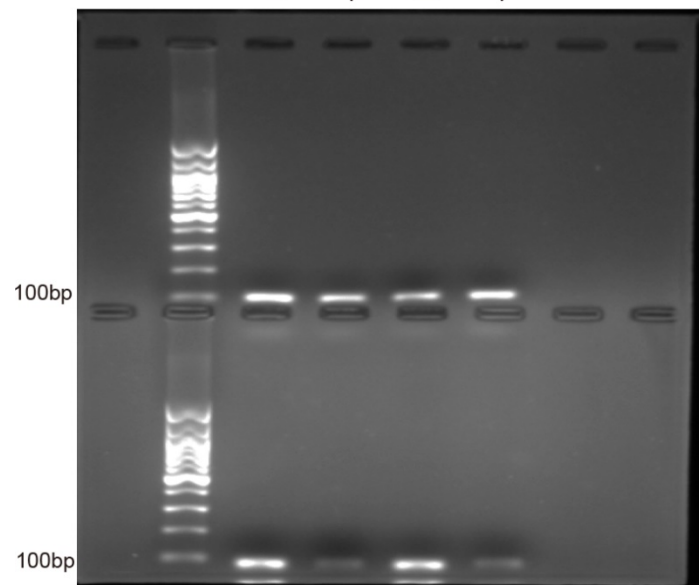

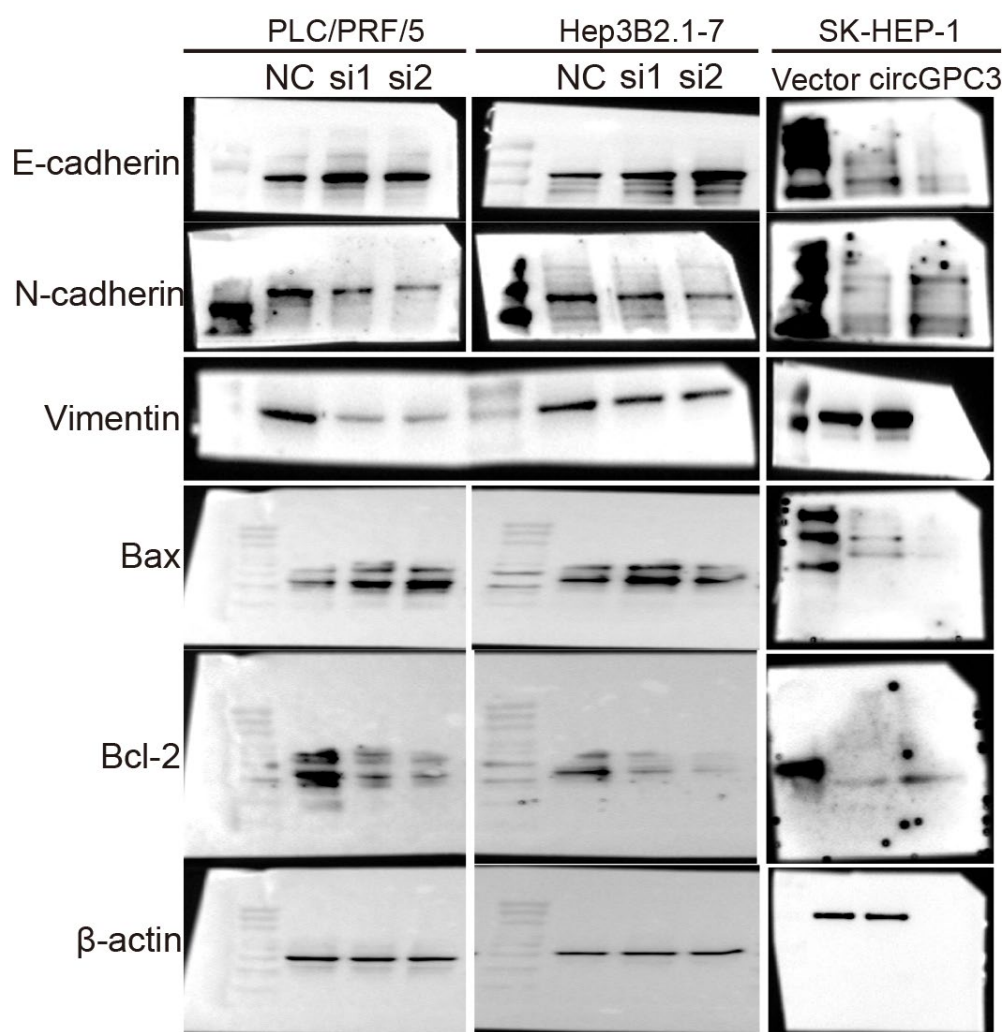

Supplement: Supplementary file 2 — Supplementary Figures. [file 41598_2024_58004_MOESM2_ESM.pdf]
